# Diagnostic and Prognostic Value of CEA and CA19-9 in Colorectal Cancer

**DOI:** 10.3390/diseases9010021

**Published:** 2021-03-17

**Authors:** Leilani Lakemeyer, Silvia Sander, Mathias Wittau, Doris Henne-Bruns, Marko Kornmann, Johannes Lemke

**Affiliations:** 1 Department of General and Visceral Surgery, University Hospital Ulm, 89081 Ulm, Germany; leilani.lakemeyer@gmail.com (L.L.); mathias.wittau@uniklinik-ulm.de (M.W.); doris.henne-bruns@uni-ulm.de (D.H.-B.); marko.kornmann@uniklinik-ulm.de (M.K.); 2 Institute of Epidemiology and Medical Biometry, University of Ulm, 89081 Ulm, Germany; silvia.sander@uni-ulm.de

**Keywords:** colorectal cancer, CEA, CA19-9, carcinoembryonic antigen, carbohydrate antigen 19-9, biomarkers, tumor markers, survival, prognosis

## Abstract

Colorectal cancer (CRC) is the third most common cancer worldwide. A diagnosis at early stages with enhanced screening methods is vital as metastases and recurrences increase mortality. The aim of this study was to analyze the tumor markers CEA and CA19-9 combined in correlation with diagnostics and prognosis. Therefore, 1487 patients with CRC who were diagnosed and treated between 2000 and 2015 at the University Hospital Ulm, Germany, were retrospectively evaluated. Overall and recurrence-free survival was analyzed in association with preoperative CEA and CA19-9 separately and combined and a multivariate analysis was performed. The 5-year overall survival was significantly shorter in patients with a CEA or CA19-9 level ≥200 compared to patients with an increased, but <200, or normal level (CEA: 69%/44%/7%; CA19-9: 66%/38%/8%). Patients with both tumor markers increased also showed a remarkably shorter 5-year survival rate (CEA+/CA19-9+: 23%). The multivariate analysis emphasizes these results (*p*-value < 0.0001). Patients with both tumor markers elevated had the shortest 5-year recurrence-free survival rate, followed by patients with either CEA or CA19-9 elevated (CEA-/CA19-9-: 79%; CEA+/CA19-9; CEA-/CA19-9+: 65%; CEA+/CA19-9+: 44%). In conclusion, measuring CEA and CA19-9 preoperatively in CRC patients is reasonable and could be useful as a prognostic factor.

## 1. Introduction

It is estimated that, every year, approximately 18.1 million patients are diagnosed with cancer worldwide and around 9.6 million patients die from their disease [1]. Colorectal cancer (CRC) remains one of the leading causes of cancer-related deaths, being the third most common malignancy, with a mortality of 608,000 every year [2]. Surgery in combination with multimodal treatment provides a cure for many CRC patients, when they are diagnosed at an early, non-metastatic tumor stage [3]. However, patients with advanced and metastatic CRC show a poor outcome, with a 5-year overall survival rate of only 19.1% reported by Sudo et al. and an even lower rate of only 8.1% reported by O’Connell et al. [4,5]. Therefore, it is becoming increasingly important to detect the disease in early stages.

For CRC, there are already many screening methods for early tumor detection available, such as the occult blood test, digital rectal exam, colonoscopy, and computed tomography [6,7]. As far as an R0 outcome is possible, a surgical resection of the solid cancer and its metastases is pursued [8,9]. Nevertheless, 10–15% of patients experience a recurrence of the disease after the radical operation, mainly within the first 18 months [8]. Again, early detection during the follow-up program is vital as a recurrence is curable when treated early by surgical resection [10]. To prevent or detect a relapse early, clinics use an intense follow-up program for 5 years after the complete resection of the tumor, which includes anamnesis, a physical examination, blood tests, including the search for tumor markers such as carcinoembryonic antigen (CEA) and carbohydrate antigen 19-9 (CA19-9), a colonoscopy, an abdominal ultrasound, a rectoscopy, computed tomography and, a chest X-ray [7].

For both detecting CRC in its early stages and for an effective follow-up program after the diagnosis of CRC, it is important to establish further knowledge and methods to improve the search for malignancies and metastases. Since tumor markers are non-invasive and cost-effective, they would be a meaningful alternative.

Since 1965, the tumor marker CEA has been known as a glycoprotein that can be detected in the blood and in cancer cells of adenocarcinomas [11,12,13]. CEA is an intracellular adhesion molecule with a half-life of 1 to 3 days that is produced in fetal gut tissue and by epithelial tumor cells, where it helps with angiogenesis [11,14]. An increased serum CEA level is found in malignancies such as colorectal, breast, gastric, lung, ovarian, and pancreatic cancer [11,12]. Nevertheless, an elevation of CEA may also be seen in many non-malignant conditions, such as cigarette smoking, alcoholism, chronic inflammatory bowel disease, diverticulitis, pancreatitis, and liver disease [15,16,17].

CA19-9 has been known since 1979 and is used more frequently for early detection of pancreatic carcinomas nowadays [18]. CA19-9 is a monoclonal antibody that is a ligand for E-Selectin [19,20]. An increase in serum CA19-9 can be found in malignant and benign processes. The tumor marker is predominantly produced by pancreatic, gastric, lung, biliary tract, and colorectal cancer. Nevertheless, patients diagnosed with liver cirrhosis, acute cholangitis, diabetes mellitus, endometriosis, or bronchiectasis also show increased levels of CA19-9 [21].

Whereas studies were presenting a sensitivity level for CEA ranging from 65% to 74% in CRC patients, CA19-9 only had sensitivity ranging from 26% to 48% [14,22,23]. Despite the low sensitivity for CA19-9 on its own, studies detected that CA19-9 correlates with the tumor marker CEA and may, therefore, improve the sensitivity of CEA [23,24,25,26,27,28]. On the other hand, the study by Bagaria et al. in 2013 showed no increase in sensitivity to CEA when both tumor markers were analyzed together [22].

Nowadays, guidelines still only recommend the use of CEA alongside other screening methods for determining prognosis, for surveillance after a curative resection, and for monitoring treatment, such as chemotherapy or radiation. Due to the low sensitivity, the use of CA19-9 on its own for detecting CRC or monitoring ongoing therapy or follow-up is not recommended [29,30,31,32,33].

The behavior and usefulness of the combination of CEA and CA19-9 for CRC patients have not yet been investigated sufficiently to make any guideline-oriented recommendations.

Since most publications evaluated either CEA or CA19-9 in correlation with CRC, this study examined the value of both tumor markers combined in diagnostics and prognosis. The aim of the study was to find an increased informative value when interpreting both tumor markers together to increase the chances of survival of patients and to optimize treatment in the future. Therefore, data of patients who were diagnosed with CRC between the years 2000 and 2015 were collected. During this time period, 1487 patients were diagnosed and treated for CRC at the University Hospital Ulm.

The behavior of the preoperatively measured tumor markers CEA and CA19-9 in correlation with clinicopathological parameters was then analyzed. Furthermore, the function and usefulness of the two markers as a predictor for overall survival and recurrence-free survival was examined and a multivariate analysis was performed.

## 2. Materials and Methods

Between 2000 and 2015, 1487 patients were diagnosed and treated with CRC in the surgical clinic at the University Hospital Ulm, Germany. Out of this patient collective, 20 patients were identified with a non-adenocarcinoma and 509 patients did not have any tumor markers documented and, therefore, had to be excluded from this study. Whereas the majority of the remaining 958 patients received surgical resection as primary treatment, 71 patients (8%) were treated with neoadjuvant therapy prior to surgery.

For all patients, a follow-up program was initiated which included documentation of life status, local recurrences or distant metastases, lymph node metastases, and tumor marker levels of CEA and CA19-9. The last update of the follow-up was made in June 2018. Further information, such as primary tumor size (pT), lymph node status (pN), existence of distant metastases (pM), grading, and Union for International Cancer Control (UICC staging), was taken from histopathological reports.

An electro-chemiluminescence immunoassay (ECLIA) was used for the in vitro quantitative determination of the tumor markers CEA and CA19-9. These two tumor markers were measured at the time of diagnosis and a few days before the surgical resection. Due to the follow-up program, the tumor markers were measured every six months for the first two years after the operation, followed by once a year for another three years. For this study, the tumor markers measured within two weeks prior to primary surgical tumor resection were of particular interest. CEA levels below 5 ng/mL were defined as normal and everything equal to or above 5 ng/mL was defined as increased levels. For CA19-9, a level below 37 U/mL was defined as normal and a level equal to or above was defined as an increased value. These cut-off values were derived from the reference levels used by the laboratory of the University Hospital Ulm.

The patient data were collected and listed with the help of Microsoft Excel 2011^®^ and were analyzed with SAS^®^ (SAS Institute, Cary, NC, USA). Qualitative data were calculated as absolute and relative frequencies, and for quantitative data, median, minimum, and maximum were used. The different tumor marker combinations were presented in association with clinicopathological parameters such as gender, age at time of diagnosis, BMI (body mass index), TNM classification, UICC stages, pathological grading, and tumor localization. Kaplan–Meier curves were used to present the overall survival and recurrence-free survival. The overall survival was defined as the time period between the date of primary diagnosis and the date of death or last surveillance. The recurrence-free survival was generated from the date of primary diagnosis until the date of the first recurrence diagnosis or death with the tumor documented as cause of death. Finally, a multivariate analysis was performed as a Cox proportional hazards ratio model to identify independent risk factors for overall survival. The results include the absolute number of patients, the hazard ratio, the 95% confidence interval, and *p*-value.

## 3. Results

### 3.1. Patient Characteristics

**Figure 1:**
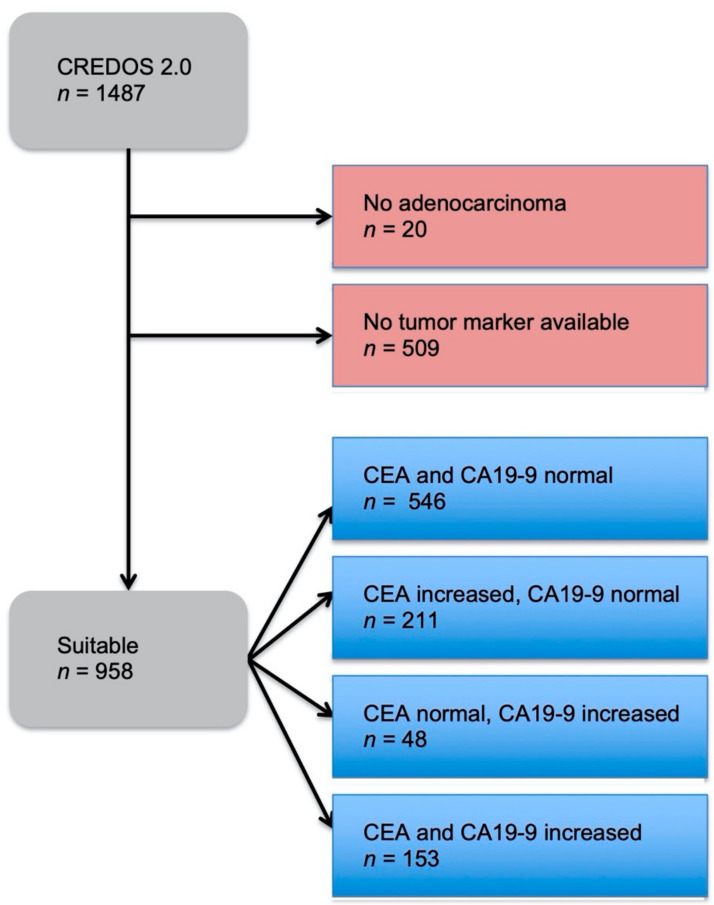
Study design of the patient collective in this study. The two red boxes represent the patients that were excluded from the study. The blue boxes represent the patients that were suitable for the study and their subdivision into four groups: both tumor markers below cut-off value, only CEA increased, only CA19-9 increased, and both tumor markers increased.

**Figure 2:**
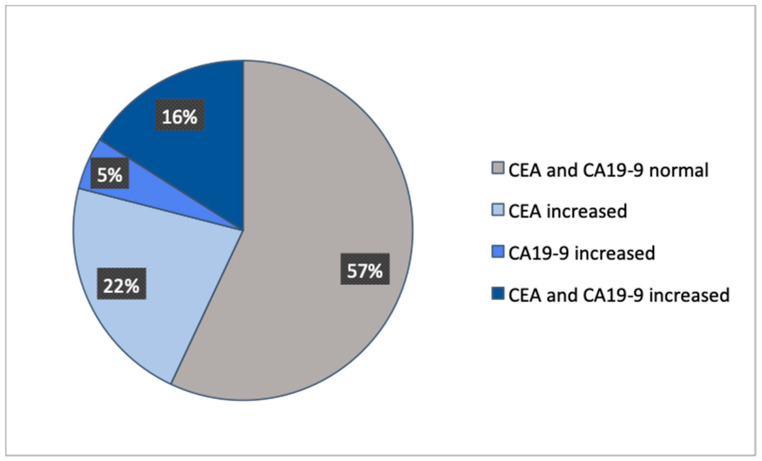
Distribution of the patient collective.

A total of 1487 patients with CRC were included in this study, in which 509 patients did not have a tumor marker documented and 20 patients were not diagnosed with an adenocarcinoma (Figure 1). Out of the remaining 958 patients, four groups were formed dependent on normal or increased preoperative CEA and CA19-9. The distribution showed that 57% had both preoperative tumor markers below the cut-off value, 22% had only CEA increased, 5% had only CA19-9 increased, and 16% had both tumor markers increased (Figure 2). These four groups were then set in correlation with patients’ characteristics, such as gender, age at time of diagnosis, BMI, tumor localization, UICC classification, and grading (Table 1).

### 3.2. Diagnostic Value of CEA and CA19-9

First of all, we analyzed the behavior of preoperative CEA and CA19-9 in correlation with patients’ characteristics to determine the possible diagnostic value of both tumor markers combined. The patients’ characteristics only showed coherence with the tumor markers dependent on the progress of the tumor. While the majority of patients in this patient collective with CRC in UICC stage I and II had both preoperative tumor markers below the cut-off value (UICC stage I + II: 72%), patients diagnosed with higher tumor stages had either only the tumor marker CEA (UICC stage IV: 32%) or both tumor markers (UICC stage IV: 42%) increased. Assessing the pathological grading in this patient collective, a slightly higher number of patients with both tumor markers elevated was shown in poorly differentiated tumors (22%) in comparison to patients with well-differentiated tumors (15%). Nevertheless, both groups still had a high number of patients with both tumor markers below the cut-off value (low grade: 59%; high grade: 47%). All other parameters did not show an association with CEA and CA19-9. Therefore, preoperative CEA and CA19-9 only have diagnostic value in evaluating staging.

### 3.3. Prognostic Value of CEA and CA19-9

To analyze the prognostic value of CEA and CA19-9, Kaplan–Meier curves were created, showing the overall survival in dependency with CEA and/or CA19-9.

**Figure 3:**
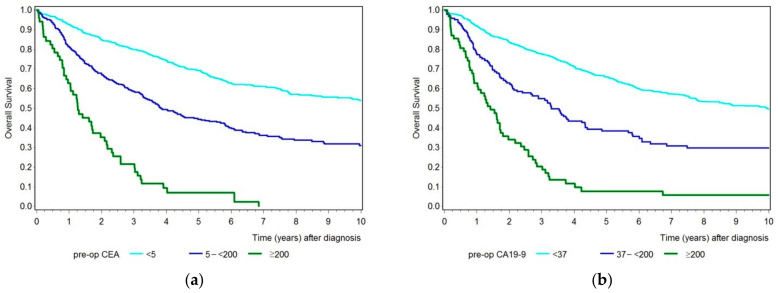
(**a**) Kaplan–Meier curves showing overall survival for patients with a preoperative CEA level below the cut-off value (turquois), with an increased CEA value between 5 and <200 ng/mL (blue), and with a CEA value ≥ 200 ng/mL (green). (**b**) Kaplan–Meier curves showing overall survival for patients with a preoperative CA19-9 level below the cut-off value (turquois), with an increased CA19-9 value between 37 and 200 U/mL (blue), and with a CA19-9 value ≥ 200 U/mL (green).

First, we compared the overall survival of patients by analyzing CEA and CA19-9 separately, dividing the patient collective into groups representing the tumor marker below the cut-off value, with an increased tumor marker but below 200, and with a tumor marker equal to and above 200 (Figure 3a,b). Both tumor markers showed a similar outcome. The higher the measured preoperative tumor marker, the lower reported patients’ survival. Whereas the 5-year survival rate (Table 2) for patients with a CEA value below 5 ng/mL was 69% (95% CI 65–73%), and for patients with a CEA value of 5 to <200 ng/mL was 44% (95% CI 38–50%), the overall survival for 5 years was only 7% (95% CI 2–17%) for patients with a CEA value equal to or above 200 ng/mL. The 5-year survival rate was 66% (95% CI 62–69%) for patients with a normal CA19-9 value, 38% (95% CI 30–47%) for patients with a CA19-9 value between 37 and below 200 U/mL, and only 8% (95% CI 3–17%) for patients with a CA19-9 value equal to and above 200 U/mL.

**Figure 4:**
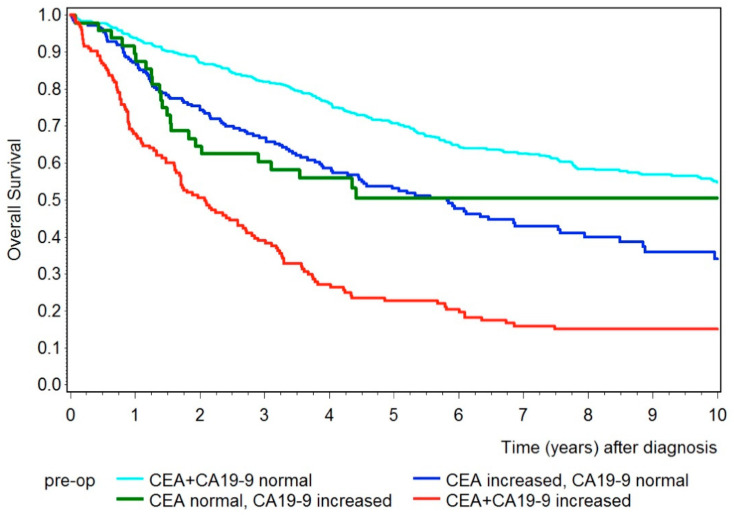
Kaplan–Meier curves showing overall survival for patients with both preoperative tumor markers below the cut-off value (turquois), with only CEA increased (blue), with only CA19-9 increased (green), and with both tumor markers increased (red).

Next, we analyzed the overall survival for CEA and CA19-9 combined, shown in Figure 4. Again, patients with either CEA or CA19-9 increased had shorter survival compared to patients with both tumor markers below the cut-off value. The 5-year survival rate for patients with both tumor markers below the cut-off value was 71% (95% CI 67–75%), 53% (95% CI 46–69%) for patients with only the tumor marker CEA elevated, and 51% (95% CI 35–64%) for patients with only the tumor marker CA19-9 elevated. However, patients with both tumor markers increased had an even shorter survival rate, with a 5-year survival rate of 23% (95% CI 16–30%).

### 3.4. Recurrence-Free Survival

**Figure 5:**
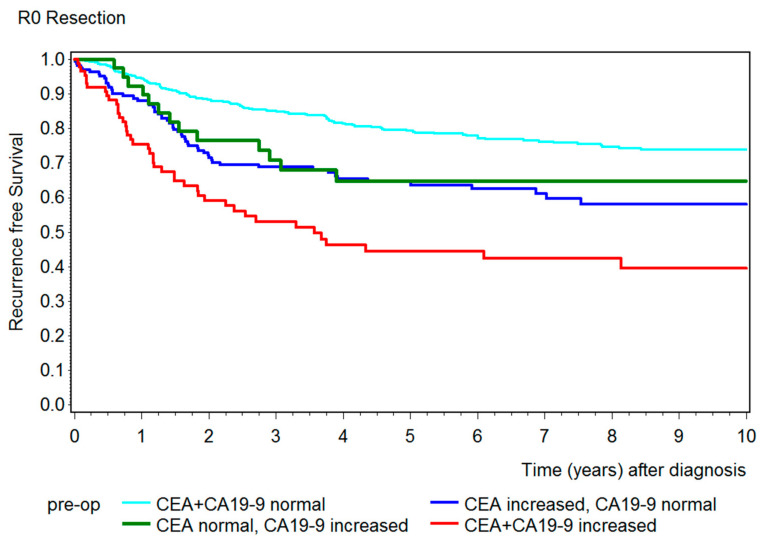
Kaplan–Meier curves showing the recurrence-free survival for patients that were R0 resected with both preoperative tumor markers below the cut-off value (turquois), with only CEA increased (blue), with only CA19-9 increased (green), and with both tumor markers increased (red).

After showing a correlation between preoperative CEA and CA19-9 and overall survival, we created a further Kaplan–Meier curve to emphasize the correlation of both tumor markers with recurrence-free survival (Figure 5). Patients who were only partially resected (R1) in the primary operation were not representative and had to be excluded from this graphic. The results were similar to the overall survival, which showed that patients with both tumor markers below the cut-off value had the best outcome and patients with both tumor markers increased had a remarkably shorter recurrence-free survival. Here, the 5-year recurrence-free survival (Table 2) for patients with both preoperative tumor markers below the cut-off value was 79% (95% CI 75–83%), 65% (95% CI: CEA: 56–72%; CA19-9: 47–78%) for patients with only the tumor marker CEA or CA19-9 elevated, and 44% (95% CI: 33–56%) for patients with both tumor markers increased.

### 3.5. Multivariate Analysis for Overall Survival

A multivariate analysis was performed, shown in Table 3, to prove the correlation between increased preoperative CEA and CA19-9 and overall survival without being influenced by other variables. Therefore, Table 3 confirms the results emphasized in Figure 4, showing both tumor markers as independent parameters for overall survival (*p*-value < 0.0001). Further independent parameters for a significantly poorer life expectancy were male gender, age at time of diagnosis, body mass index below 25, UICC III/IV, G3/4, and R1 resection.

## 4. Discussion

The usefulness of CEA and CA19-9 in screening, follow-up after diagnosis, and monitoring treatment has been explored in CRC patients since their discovery. Until now, guidelines recommended only the use of CEA for determining prognosis, surveillance after a curative resection, and monitoring treatment. CA19-9 is still not recommended as a useful marker in CRC patients [29,30,31,32,33]. The behavior and usefulness of the combination of CEA and CA19-9 have not yet been investigated sufficiently to make any guideline-oriented recommendations.

This study was able to provide further information about the value of measuring both tumor markers preoperatively. Nevertheless, there remain limitations to this study, such as the remaining bias as this study only included patients treated in a surgical clinic. Since this article is based on a retrospective study, we recommend further prospective studies including patients from different faculties.

This study clearly shows that even both tumor markers combined are not eligible to replace any of the existing screening methods. Only patients diagnosed with stage IV CRC showed a remarkable association with an elevated CEA and CA19-9 level. The same results were shown in studies also examining CEA and CA19-9 in combination and their association with UICC and TNM stages [23,24,34].

Nevertheless, the measurement of preoperative CEA and CA19-9 level remains important for further monitoring and treatment. As mentioned before, guidelines recommend CEA as a useful predictor of overall survival [29,31,35]. This study was able to underline this assertion, as we were able to present CEA as a reliable predictor for overall survival. We were able to show a shorter 5-year survival rate of 44% in patients with an elevated CEA compared to patients with a normal CEA (69%) and a remarkably shorter 5-year survival rate of only 7% in patients with a CEA level over 200 ng/mL. Many studies presented a similar outcome using the same cut-off value of 5 ng/mL for the tumor marker CEA [25,36,37,38,39,40,41,42]. On the other hand, studies that examined a patient collective with only stage IV CRC did not show a correlation between CEA and overall survival [26,34,43,44]. In patients with a stage IV CRC, Ishizuka et al. only found a significant difference between the overall survival curves when setting the cut-off value at 150 ng/mL for the preoperative serum CEA level. Park et al. examined different cut-off values for preoperative CEA in 2005 and were able to demonstrate a significantly worse overall survival rate in patients using a cut-off value of 17 ng/mL [45].

Unlike CEA, guidelines do not recommend the use of CA19-9 for determining a prognosis [29,30,35]. Nevertheless, this study was able to demonstrate the similar significance of CA19-9 as a predictor of survival compared to the tumor marker CEA. Patients with increased preoperative CA19-9 had a significantly poorer 5-year overall survival rate of 38% compared to patients with a normal CA19-9 level (66%). Again, patients with a CA19-9 level of over 200 U/mL had a remarkably shorter 5-year survival rate of only 8%. Many other studies were able to present similar results using slightly different cut-off values between 31 and 37 U/mL [25,26,27,28,34,36,37,38,39,40,46]. Again, a couple of studies analyzing only patients with stage IV CRC did not show a correlation between CA19-9 and overall survival [43,44]. Ishizuka et al. only found a significant difference in overall survival when scaling the cut-off value up to 200 U/mL [43]. In 1994, Ueda et al. had already presented an even shorter survival outcome where all patients with a CA19-9 value over 160 U/mL had died within 1.25 years [25]. Lu et al. compared overall survival with different cut-off values such as 35, 100, and 200 U/mL. In this study, no significant difference appeared between the different cut-off values. Already, a cut-off value of 35 U/mL had shown significantly shorter survival for patients with CRC [27].

Until now, only a few studies have analyzed overall survival in correlation with CEA and CA19-9 combined. This study presents a remarkably shorter 5-year survival rate of only 23% for patients with both tumor markers elevated compared to patients with either CEA or CA19-9 (CEA:53%, CA19-9: 51%) or no tumor marker (71%) increased. In addition to a shorter outcome in patients with both tumor markers increased, the results presented by Thomsen et al. and Shin et al. showed equally short overall survival in patients with only the tumor marker CA19-9 increased [28,36]. Graziosi et al. only formed two groups including patients with both tumor markers elevated and with either CEA or CA19-9 elevated. Here, patients with both tumor markers increased had significantly shorter survival [40]. However, a study by Shibutani et al. also included patients with either CEA or CA19-9 elevated in one group. There was no significant difference in survival between patients with both tumor markers below the cut-off value and patients with only one tumor marker elevated. On the other hand, patients with both tumor markers elevated had significantly shorter survival [38].

Further, in addition to underlining the usefulness of CEA and CA19-9 combined in predicting overall survival, this study showed the great informative value of both tumor markers in recurrence-free survival. Again, the 5-year recurrence-free survival rate of patients with preoperative tumor markers elevated was remarkably shorter (44%) than in patients with only one tumor marker increased (65%) or with both tumor markers below the cut-off value (79%). A couple of studies presented the same results, with similar recurrence-free survival rates [28,47]. Whereas Zhang et al. and Shibutani et al. also presented a much shorter recurrence-free survival rate for patients with both tumor markers increased, patients with only one or no tumor markers increased showed a similar outcome in terms of recurrence-free survival [34,38].

## 5. Conclusions

After evaluating both preoperative tumor markers CEA and CA19-9 in colorectal cancer patients in correlation with patients’ characteristics, we do not recommend the use of CEA and CA19-9 in the screening program. The study did not establish any reliable association between the level of both tumor markers and gender, age at time of diagnosis, BMI, TNM classification, UICC stages, grading, and tumor localization. Therefore, the currently used screening methods for early detection, such as occult blood test, digital rectal exam, colonoscopy, and computed tomography, should remain the recommended guidelines.

After analyzing the preoperative tumor markers CEA and CA19-9 separately and combined in correlation with overall survival and recurrence-free survival, we come to the conclusion that both biomarkers give significant prognostic information. In this study, increased CEA and CA19-9 levels evaluated separately already showed significant differences in overall survival. Then, when analyzing both tumor markers in combination in overall survival and recurrence-free survival, an even more significant result emerged, showing the correlation of increasing tumor markers resulting in lower life expectancy and recurrence-free survival.

While diagnostic benefits are not observed, we do recommend measuring both tumor markers CEA and CA19-9 for prognostic purposes before performing primary surgery.

Since the combination of CEA and CA19-9 acts as an independent prognostic marker for survival, we suggest considering a more aggressive therapy in patients diagnosed with advanced CRC, however low their CEA and CA19-9 levels, given their superior prognosis.

## Figures and Tables

**Table 1:** Patients’ characteristics.

Patients’ Characteristics									
Parameter	CEA− CA19-9−		CEA+ CA19-9−		CEA− CA19-9+		CEA+ CA19-9+		Total
	*n* = 546	in %	*n* = 211	in %	*n* = 48	in %	*n* = 153	in %	*n* = 958
Gender									
Male	354	59	130	21	22	4	95	16	601
Female	192	54	81	23	26	7	58	16	357
Age at Time of Diagnosis									
<65 years	229	57	106	26	13	3	56	14	404
≥65 years	317	57	105	19	35	6	97	18	554
Median	67		64		68		67		67
(Min:Max)	(20:93)		(29:89)		(34:89)		(36:95)		(20:95)
Body Mass Index									
<25	189	52	95	26	25	7	55	15	364
≥25	351	61	114	20	21	3	92	16	578
N/A	6	37	2	12	2	13	6	38	16
Tumor Localization									
Right Colon	129	48	61	23	21	8	57	21	268
Left Colon	106	54	48	24	7	4	35	18	196
Rectum	282	65	87	20	18	4	46	11	433
C18.8/C18.9	29	47	15	25	2	3	15	25	61
UICC Classification									
UICC I + II	328	72	82	18	16	4	28	6	454
UICC III	159	62	48	19	23	9	26	10	256
UICC IV	52	22	77	32	9	4	99	42	237
N/A	7	64	4	36	0	0	0	0	11
Grading									
Low grade	419	59	154	22	28	4	104	15	705
High grade	96	47	43	21	20	10	46	22	205
GX	31	65	14	29	0	0	3	6	48

CEA = carcinoembryonic antigen; CA 19-9 = carbohydrate antigen 19-9; − = below cut-off value; + = increased; *n* = absolute number of patients; N/A = no data available; right colon = including vermiform appendix, cecum, ascending colon, hepatic flexure, transverse colon; left colon = including splenic flexure, descending colon, sigmoid colon; C18.8 = malignant neoplasm of overlapping sites of colon; C18.9 = malignant neoplasm of colon, unspecified; UICC = Union for International Cancer Control G = pathological grading; low grade = G1/G2; high grade = G3/G4; GX = undetermined grade.

**Table 2:** Impact of CEA and CA19-9 on survival.

Group	*n*	5y-OSR (95% CI)	5y-RFS (95% CI)
CEA (in ng/mL)			
<5	635	69% (65%; 73%)	-
5–<200	349	44% (38%; 50%)	-
≥200	51	7% (2%; 17%)	-
CA19-9 (in U/mL)			
<37	758	66% (62%; 69%)	-
37–<200	140	38% (30%; 47%)	-
≥200	62	8% (3%; 17%)	-
CEA and CA19-9 combined			
CEA− CA19-9−	544	71% (67%; 75%)	79% (75%; 83%)
CEA+ CA19-9−	212	53% (46%; 60%)	65% (56%; 72%)
CEA− CA19-9+	48	51% (35%; 64%)	65% (47%; 78%)
CEA+ CA19-9+	153	23% (16%; 30%)	44% (33%; 56%)

5y-OSR = 5-year overall survival rate; 5y-RFS = 5-year recurrence free survival rate; CI = confidence interval; CEA = carcinoembryonic antigen; CA19-9 = carbohydrate antigen 19-9; + = increased; − = below cut-off value.

**Table 3:** Multivariate analysis for overall survival as the Cox proportional hazards model.

	Patients	HR	95% CI	*p*-Value
Gender				
Female	575	1.00		0.043
Male	892	1.228	1.0–1.5	
Age at diagnosis				
Unit = 1	1467	1.034	1.0–1.05	<0.0001
Body Mass Index				
≥25	846	1.00		0.018
<25	538	1.254	1.0–1.5	
UICC stage				
UICC I/II	704	1.00		<0.0001
UICC III/IV	733	1.737	1.4–2.2	
Grading				
Low grade	1043	1.00		0.002
High grade	332	1.408	1.1–1.7	
Preop tumor markers				
CEA−, CA19-9−	544	1.00		<0.0001
CEA+, CA 19-9−	212	1.343	1.1–1.7	
CEA−, CA 19-9+	48	1.263	0.8–1.9	
CEA+, CA19-9+	153	1.961	1.5–2.5	
R-status				
R0	1224	1.00		<0.0001
R1	243	3.596	2.8–4.6	

CI = confidence interval; HR = hazard ratio; UICC = Union for International Cancer Control; CEA = carcinoembryonic antigen; CA19-9 = carbohydrate antigen 19-9; + = increased; low grade = G1/G2; high grade = G3/G4; − = below cut-off value; R-status = status of resection; R0 = fully resected primary tumor; R1 = partially resected primary tumor.

## Data Availability

Supporting data was not included in our study.
